# Outcome of anastomotic urethroplasty in traumatic stricture (distraction defect) of posterior urethra in boys

**DOI:** 10.1080/2090598X.2020.1716294

**Published:** 2020-02-09

**Authors:** Ghulam Mujtaba Zafar, Sikandar Hayat, Javeria Amin, Fawad Humayun

**Affiliations:** Pediatric Urology, The Children’s Hospital and Institute of Child Health, Lahore, Pakistan

**Keywords:** Urethroplasty, urethral strictures, distraction defect, pubectomy, boys

## Abstract

**Objective:**

To report the outcomes of operative management of traumatic posterior urethral distraction defect in boys at our Centre, as traumatic posterior urethral stricture in children is a rare condition that presents a major surgical challenge to the paediatric urologist and consensus on the optimal treatment of these strictures in children has not been reached.

**Patients and methods:**

We retrospectively analysed our data from July 2013 to June 2018. All boys aged ≤16 years with traumatic posterior bulbo-prostatic obliteration (distraction defect) were included. Initial suprapubic cystostomy and delayed definite anastomotic urethroplasty was done in all the boys. The boys were evaluated preoperatively with a retrograde urethrogram and simultaneous voiding cystourethrogram, as well as cystourethroscopy.

**Results:**

A total of 38 boys, with posterior urethral distraction defect, were divided into primary and redo surgery groups. The primary group comprised 34 boys who were operated upon for the first time. A perineal approach with development of an inter-crural space was done in 12 boys and along with an inferior pubectomy in 19 boys. Three boys in the primary group needed a transpubic approach due to a longer defect. In the redo group, there were six boys, of which four were operated initially outside our hospital, while two were our own unsuccessful urethroplasties. In the redo group, a perineal approach with inferior pubectomy was done in two boys and a transpubic urethroplasty in the remaining four boys. The success rate of anastomotic urethroplasty without any ancillary procedures was 81.5% (strict criterion), while the overall success rate was 94.7% (permissible criterion, which included boys who were managed later with direct vision internal urethrotomy and dilatation).

**Conclusion:**

The ideal treatment of post-traumatic posterior urethral defect/strictures in boys is tension-free bulbo-prostatic anastomosis. This was done using a transperineal approach in most of the boys, but a few required a transpubic approach, with good results.

**Abbreviations:**

DVIU: direct vision internal urethrotomy; SPC: suprapubic cystostomy; SUI: stress urinary incontinence

## Introduction

Traumatic posterior urethral stricture in children is a rare condition, but presents a major surgical challenge to paediatric urologists. Paediatric pelvic fracture after blunt trauma has an incidence of 2.4–4.6%. Of these, only 4.2% are associated with urethral injuries [1]. The mechanism of traumatic posterior urethral injury is unique in which there is complete or partial urethral rupture with separation and malalignment of the two ends, resulting in a distraction defect. While, in true urethral stricture there is continual obliteration of the urethral lumen. Complete urethral disruption and distraction defect is more common in children as compared to adults due to severe displacement of the prostatic urethra off the pelvic floor [2]. Access to the posterior urethra and management of distraction defect in children is difficult for many reasons. Firstly, children have immature pelvic bones and unstable fractures associated with severely displaced prostatic urethras. Secondly, due to relative intra-abdominal position of a child’s bladder there is high incidence of simultaneous bladder neck and sphincter complex injury along with urethral trauma [3]. Lastly, children have smaller pelvic confines, smaller urethral calibre, and greater tissue fragility [4]. Thus, considering all these differences, the management of traumatic posterior urethral strictures in children is more challenging and needs perineal anastomotic urethroplasty in most cases, while a more extensive transpubic approach is needed in some cases with large gaps [2].

In the present study, we present our experience of the management of boys with traumatic posterior urethral stricture/distraction defects.

## Patients and methods

The data were reviewed from July 2013 to June 2018. All boys aged ≤16 years with traumatic distraction defect (stricture) were included in the study. Stricture following corrective surgery (hypospadias, epispadias, and exstrophy repair) and patients with incomplete medical records were excluded. All the boys initially underwent suprapubic cystostomy (SPC) followed by delayed urethroplasty. Some additional procedures before definite urethroplasty were performed in a few boys. ‘Rail-roading’ and catheter placement was done by an adult urologist in two boys. In four boys, unsuccessful end-to-end urethroplasty had already been done before referral. A groin flap had been applied by a plastic surgeon in one boy with massive trauma who lost both his testis and scrotum. Three boys underwent laparotomy and a diverting colostomy for associated gut injuries; one of them developed recto-urethral fistula later in addition to stricture of the urethra.

All the boys were evaluated preoperatively with a retrograde urethrogram and simultaneous voiding cystourethrogram (‘up-and-down-o-gram’) to assess the length of the obliteration and status of the bladder neck. We used urethroscopy and cystourethroscopy through the SPC tract before definite repair to assess the quality of the urethral mucosa, the gap between the two healthy ends of the urethra, as well as the bladder neck. Absence of UTI was ensured before urethroplasty. Parents were informed and counselled regarding outcomes and possible complications.

### Operative technique

**Figure 1.**
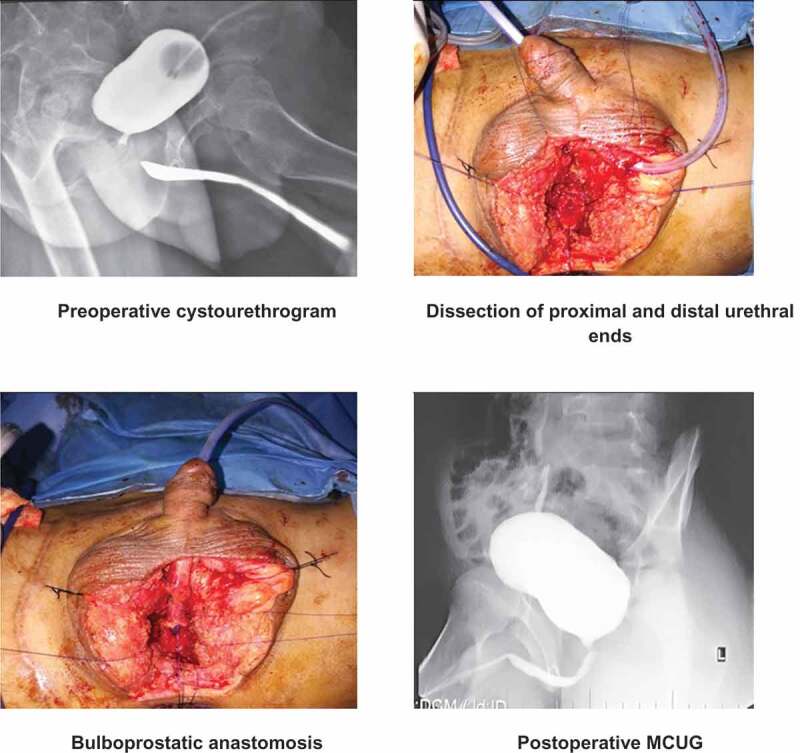
Transperineal urethroplasty.

All boys were operated upon under general anaesthesia in standard lithotomy position with proper leg support. The procedure started with a perineal inverted ‘Y’ incision on the median raphe. The bulbar urethra was exposed by incising the bulbospongiosus muscles and dissecting proximally until the obliterated segment. The fibrous tissue (defect/stricture) was completely excised. The bulbar urethra was mobilised distally until the peno-scrotal junction to achieve adequate length. Then antegrade flexible cystourethroscopy was performed through the SPC tract until the blind distal end of the posterior urethra was identified. It was opened and spatulated at the 12 O’clock position. The gap between the proximal and distal ends of the urethra was measured using a ruler. The bulbar urethra was spatulated at the 6 O’clock position and then a mucosa-to-mucosa anastomosis was made with 6/0 or 5/0 polydioxanone (PDS) suture over a silicone Foley catheter (Figure 1).

**Figure 2.**
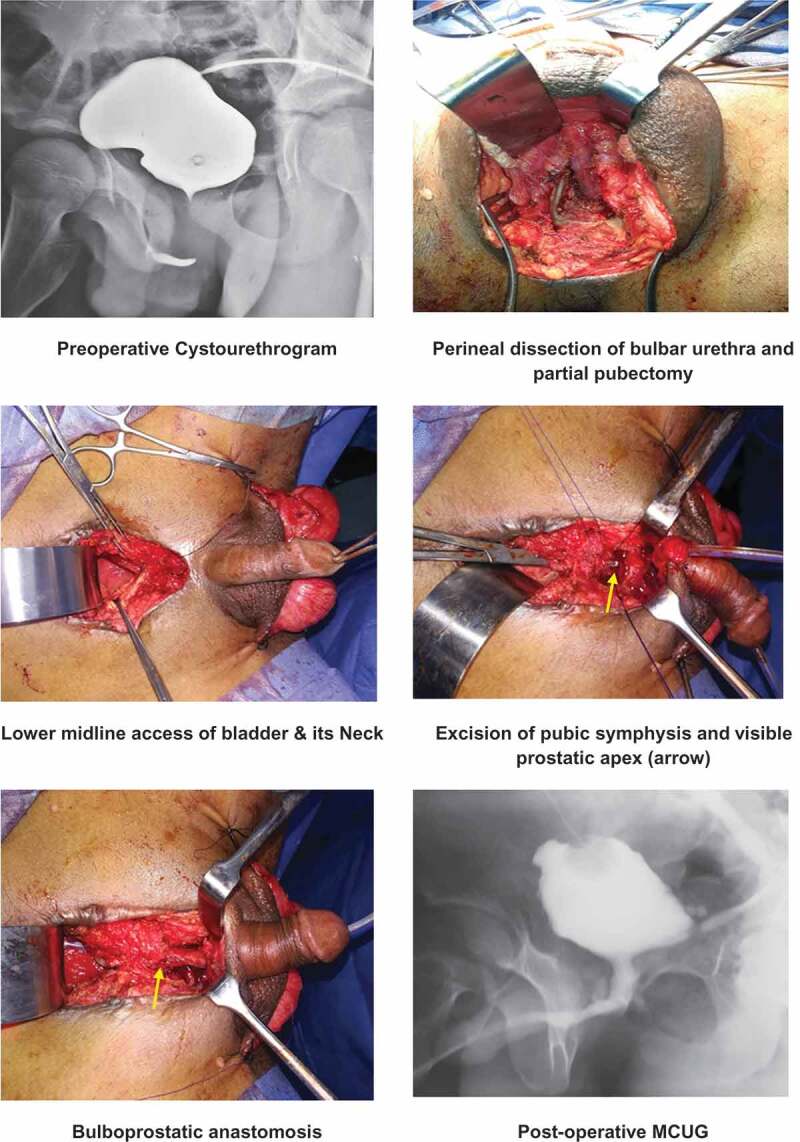
Transpubic urethroplasty.

Where a tension-free anastomosis was not possible, the inter-crural membrane was divided to create an inter-corporeal space to complete the anastomosis. In cases where the dissection was not sufficient to bridge the gap, a partial inferior pubectomy was done to complete the repair. If an adequate tension-free anastomos was not possible after both of the above mentioned manoeuvres, we proceeded with transpubic urethroplasty. In which the bladder was approached through a lower abdominal midline incision, the bladder neck and prostate exposed by removing part of the pubic symphysis, and then the bulbo-prostatic anastomosis made (Figure 2).

The SPC was kept *in situ* for adequate drainage and the urethral catheter as a stent. Aperi-catheter urethrogram was taken after 6 weeks. In cases where there was no leakage of contrast at the anastomotic site, we removed the urethral catheter and clamped the SPC. When the boy voided with good stream and without difficulty, the SPC was removed. When there was peri-catheter leakage on the urethrogram, we repeated it after 2 weeks and then removed the urethral catheter and SPC, as previously described.

The success of the procedure was measured by strict and permissible criteria. The ‘strict criterion’ was the immediate success rate without any ancillary procedure, e.g. direct vision internal urethrotomy (DVIU) or dilatation. The ‘permissible criterion’ was the ultimate success rate in which a single DIVU followed by intermittent dilatation was done after the anastomotic urethroplasty. Similar categorisation of outcomes was reported by El-Sheikh et al. [4] and designated as initial and ultimate success, and by Ali et al. [5] who also used the terms, strict and permissive criteria of success.

The boys were followed-up at 3 and 6 months, and then yearly. They were evaluated for urinary stream along with uroflowmetry, urine routine examination, and ultrasonography of the kidneys, ureters and bladder with residual urine assessment. If there was any voiding difficulty, decrease in flow rate, changes in upper urinary tract or residual urine in the bladder, radiological evaluation was repeated for anastomotic stricture or recurrence. Recurrent strictures were managed by DVIU followed by intermittent dilatation.

## Results

**Figure 3.**
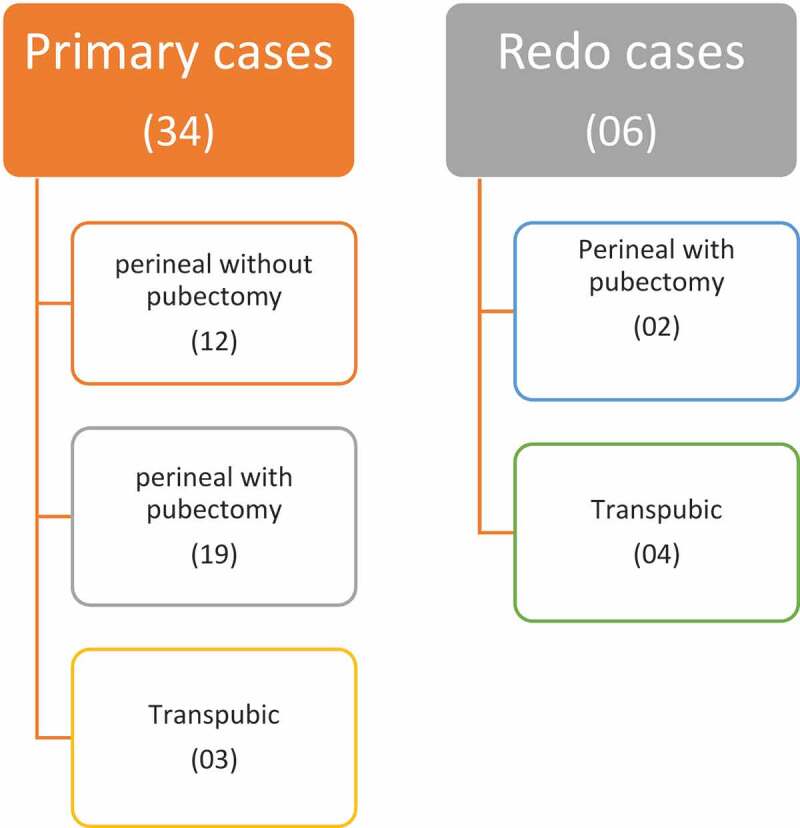
Operative techniques.

We operated on 38 boys with traumatic posterior urethral strictures who were divided into primary and redo surgery groups. The primary group comprised 34 boys who were operated upon for the first time and the urethroplasty was successful in 32 boys (permissible criterion). A perineal approach with development of the inter-crural space was used in 12 (31.57%) boys and along with an inferior pubectomy in 19 (50%) (Figure 3). A re-rooting of the corpora was done in one boy. Three boys in the primary group needed a transpubic approach due to a long gap. Two boys did not void after removal of the urethral catheter and were managed in the redo group. All 32 patients remained on follow-up and four of them developed anastomotic strictures at 0.5, 1, 2, and 4 years. All four were managed by DVIU followed by dilatation.

There were six boys in redo group; four were referred to us after unsuccessful urethroplasty, while two of them were our own failed urethroplasties. The operative techniques applied in the redo group were perineal approach with inferior pubectomy in two boys and a transpubic approach in the remaining four boys (Figure 3). All redo urethroplasties were successful except for one boy who developed voiding symptoms and partial narrowing at the anastomotic site 1 year later, and he was managed with DVIU.

**Figure 4.**
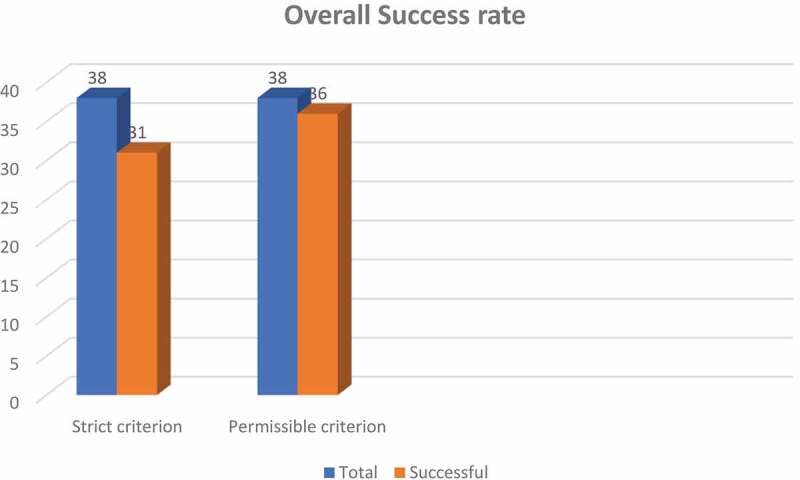
Overall success rate.

Our success rate of anastomotic urethroplasty by the strict criterion was 81.5%.The overall success rate of 94.7% was achieved when applying the permissible criterion (Figure 4). The mean (range) follow-up was 3.5 (0.5–4) years.

**Figure 5.**
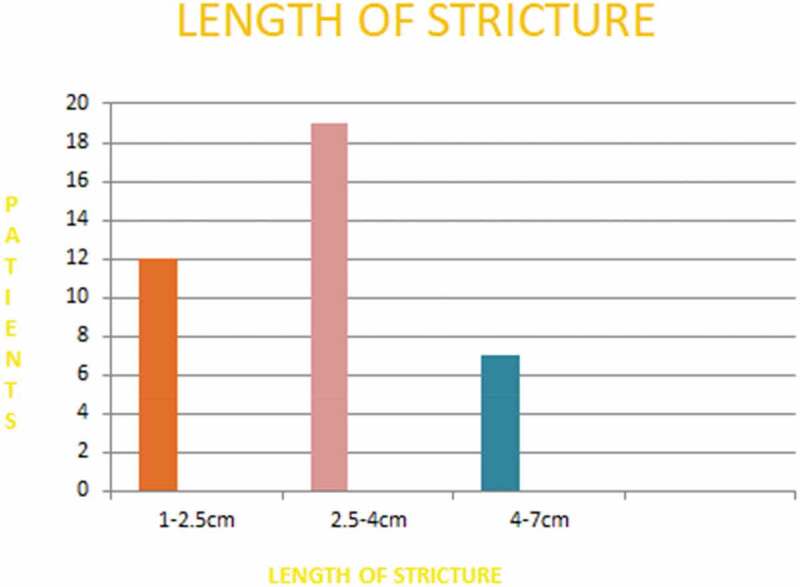
Length of strictures.

The mean (range) age of the boys was 7.92 (2–16) years. The mean gap or length of the stricture was 2.6 cm with a range of 1.0–2.5 cm in 12 boys and 2.6–4 cm in 19 boys, while seven boys had long strictures of >4 cm (range 4.1–7 cm) (Figure 5). The mean (range) hospital stay was 3 (2–5) days.

One boy had an iatrogenic rectal injury during surgery, which was repaired at the same time and fully recovered. The postoperative continence level was satisfactory, as most of the boys were continent except for stress urinary incontinence (SUI) in 11 boys during the initial few months. Of those 11 boys, 10 improved with conservative management, but one boy has persistent day time SUI off and on even after 2 years.

Of the 18 children who have reached puberty and can inform us about erections, five had evidence of erectile dysfunction.

## Discussion

Posterior urethral distraction defects in children are complex and difficult to manage. Many principles and techniques used in the repair of these urethral strictures in children are similar to those used in adults but in fact the severity, complexity, and nature of tissue are different in children. A consensus on the optimal treatment of these traumatic distraction defects in children is yet to be reached.

Early vs delayed definite repair is a debated topic and there are proponents as well as opponents on both sides. Koraitim [6] critically analysed published data over 50 years and concluded that primary suturing of the disrupted urethral ends has the greatest complication rates of UI and erectile dysfunction (21% and 56%, respectively). Mouraviev et al [7] compared early re-alignment vs delayed urethroplasty of distraction defects in 96 patients, and concluded that early realignment may provide better outcome in terms of stricture development (49% vs 100%), erectile dysfunction (33.6% vs 42.1%), and UI (17.7% vs 24.9%). But these were selected patients with milder injuries and only 19% had prostatic or membranous urethral disruptions. Based on these references, we used initial SPC and delayed definite repair in all our cases. We prefer this delayed approach due to the anticipated excessive blood loss or haematoma formation, potential instability, friability of traumatised tissues, and concurrent injuries in early repair.

Preoperative assessment of the length of the stricture and competence of the bladder neck is crucial for selection of the requisite surgical approach, as well for prognosis of the procedure and continence. Although an antegrade and retrograde contrast study (‘up-and-down-o-gram’) is done in every case, it does not give complete information in all patients, specifically when the bladder neck does not open and the posterior urethra is not outlined, giving a false impression of a long defect/stricture. It may underestimate the gap when the prostatic urethra is connected to a contrast-filled urinoma, which is not a true urethra, and anastomosis with this cavity will lead to failure or recurrence [8]. To overcome these technical problems, we perform preoperative urethroscopy and cystourethroscopy through the SPC tract in all children. This is valuable to visualise the bladder neck and posterior urethra, especially where the bladder neck does not open and the posterior urethra is not outlined on radiological evaluation. We found patent a bladder neck and posterior urethra in all such patients. This has been highlighted by other authors [2,8].

We did a simple perineal urethroplasty with separation of the corporeal bodies in 12 (31.57%) boys with a mean (range) defect/stricture of 2 (1.0–2.5) cm and partial inferior pubectomy in 19 (50%) boys bridging a mean (range) gap of 3.5 (2.6–4) cm. Orabi et al [2] presented a series of 50 patients in which perineal anastomosis was performed without inferior pubectomy in 40 patients and combined inferior pubectomy in only three patients. They were able to bridge a mean (range) gap of 1.5 (0.5–4.0) cms using a perineal approach with an overall success rate of 94%. El-Sheikh et al [4] reported on 15 patients, all treated by perineal urethroplasty and they covered gaps of up to 6 cm. Al-Rifael et al [9] reported on a study of 20 children, perineal urethroplasty was performed in four, with the remaining 16 undergoing a transpubic urethroplasty, with a success rate of 100% and 62.5%, respectively. In a study by Aggarwal et al. [10], perineal urethroplasty was possible in 10 of 23 patients, with mean gap of 3 cm. Similar recommendations were given by Podestá [11] that a distraction defect of ≥3 cm should be treated by a combined perineo-abdominal transpubic approach. We were able to perform end-to-end transperineal anastomosis in a stricture/gap of up to 4 cm. This difference in bridging the gap using a perineal approach may due to the availability of a longer or more elastic bulbar urethra in some children. Koraitim [12] proposed that the length of the bulbar urethra and its ratio to defect/stricture may help to predict the optimal approach for urethroplasty. Bulbo–prostatic urethral gaps shorter than one-third of the bulbar urethral length can usually be corrected using a simple perineal approach, while longer defects necessitate a transpubic urethroplasty. Sekhon et al [1] supported the same idea by calculating the gapometry score (G/U index), defined as the length of urethral gap divided by the bulbar urethral length. He concluded that a preoperative G/U index of 0.44 correlates with a simple perineal urethroplasty, whereas an index of >0.87 indicates the likelihood of needing a more elaborated transpubic approach. Pfalzgraf et al [13] presented a series of 17 patients and performed the excision with a perineal anastomosis in eight (47%) patients with strictures of ≤1.0 cm, while buccal mucosal onlay/inlay graft urethroplasty was performed in the remaining nine (53%) cases with strictures of >1 cm. But they included penile stricture and post-irradiation strictures in their series as well.

In the present study, transpubic urethroplasty was performed in seven boys (18.42%) who had long distraction defects of >4 cm (range 4.1–7.0 cm) and all were successful without significant haemorrhage, gait abnormality, hernia or chronic pain. Patil [14] followed five of 30 patients for 7–10 years after transpubic urethroplasty and found all competent and continent. Das et al [15] operated on 10 children using transpubic approach with 100% success rate. Basiri et al [16] reported symphysiotomy in 10 children (both boys and girls); all were successful and continent without any complications. Podesta and Podesta [17] did a comparative study of perineal vs transpubic urethroplasty for traumatic posterior urethral distraction defect in children and found a stricture-free rate of 84% for the perineal approach and 100% in those who underwent transpubic urethroplasty.

Historically, the one-stage Badenoch pull-through procedure of the bulbar urethra was proposed for a short stricture [18]. While for longer strictures the advice was to manage them by transpubic urethroplasty [19]. Koraitim [12] reported that transpubic procedures are more commonly performed in children because of a shorter bulbar urethra.

In the present study, stricture recurrence or more precisely anastomotic stenosis was seen in five (13%) boys, four in the primary group and one in the redo group. These anastomotic strictures developed in boys who voided well initially and then presented with obstructive symptoms at 0.5–4 years after their urethroplasty. All these were managed successfully with DVIU. The published incidence of re-stricture is 5–19%. Rourke et al [20] reported recurrence in 6% and Andrich et al [21] reported a 14% re-stricture rate at 15 years of follow-up. However, recurrence depends on multiple factors, e.g. the initial length of the stricture, underlying aetiology, prior urethral intervention, spongiofibrosis and para-urethral abscess [22,23]. Helmy et al [24] published a series of 65 patients, all managed by perineal urethroplasty. They reported recurrence in seven cases, five were managed endoscopically and two under went redo urethroplasty.

The postoperative UI is usually SUI and improves with time except in patients who have bladder neck injury. In our present series, 11 boys had SUI, of which 10 became dry in a few months except for one boy who still has off and on daytime SUI at 2 years of follow-up. Das et al [15] and Singla et al [25] reported excellent continence results after perineal and transpubic urethroplasty. Podesta and Podesta [17] reported nine of 49 patients had UI after urethroplasty, six of them had SUI and one had total UI. UI is most likely due to the severity of the pelvic fracture and associated bladder neck injury rather surgical trauma.

Our present study has some limitations, e.g. urinary continence was not measured with validated instruments. Erectile dysfunction was not addressed objectively and there was a short median follow-up. We subjectively evaluated few boys who were older and able to inform us about their sexual function (erection status). The strength of the present study is that all the boys were operated by a single surgeon, which alleviates operative bias.

## Conclusion

The ideal treatment of traumatic posterior urethral strictures in boys is tension-free bulbo-prostatic anastomosis. This can be done using a transperineal approach in most of cases with good results. A few patients will need a more extensive transpubic approach to bridge a large gap/stricture (>4 cm). This is contrary to the previous perception that most children with posterior urethral strictures require a transpubic approach.

## Recommendations

Adequate preoperative assessment with simultaneous ‘up-and-down-o-gram’ is crucial for the accurate estimation of the defect and thus choice of surgical procedure. All patients should be started using a perineal approach. A transpubic procedure should be done only if a tension-free anastomosis cannot be performed through the perineum.

## Submission declaration

This is declared that article is not submitted elsewhere for publications.

## References

[1] Sekhon V, Kudchadkar SJ, Raj A, et al. Radiographic gapometry score: a simple predictor for surgical approach in pediatric traumatic posterior urethral strictures. J Pediatric Urol. 2017;13:624e1–624 e5. 10.1016/j.jpurol.2017.05.016 28687410

[2] Orabi S, Badaway H, Saad A, et al. Post-traumatic posterior urethral strictures in children: how to achieve a successful repair. J Pediatric Urol. 2008;4:290–294. 10.1016/j.jpurol.2008.01.209 18644532

[3] Nerli RB, Koura AC, Ravish IR, et al. Posterior urethral injury in male children: long-term follow up. J Pediatric Urol. 2008;4:154–159. 10.1016/j.jpurol.2007.11.002 18631914

[4] El-Sheikh MG, Zaida AM, Sadek SZ, et al. Pediatric and adolescent transperineal anastomotic urethroplasty. J Pediatic Urol. 2008;4:333–336. 10.1016/j.jpurol.2008.04.008 18790414

[5] Ali S, Shahnawaz SI, Baloch MU, et al. Delayed single stage perineal posterior urethroplasty. J Coll Physicians Surg Pak. 2015;25:438–442. 26100998

[6] Koraitim MM. Pelvic fracture urethral injury: the unresolved controversy. J Urol. 1999;161:1433–1441. 10210368

[7] Mouraviev VB, Coburn M, Santucci RA. The treatment of posterior urethral disruption associated with pelvic fractures: comparative experience of early versus delayed urethroplasty. J Urol. 2005;173:873–876. 15711301 10.1097/01.ju.0000152145.33215.36

[8] Ranjan P, Asari MS, Singh M, et al. Post-traumatic urethral stricture in children: what we have learned over the years? J Pediatric Urol. 2012;8:234–239. 10.1016/j.jpurol.2011.06.004 21764640

[9] Al-Rifael MA, Gaafar S, Abdel-Rehman M. Management of posterior urethral strictures secondary to pelvic fractures in children. J Urol. 1991;145:353–356. 1988730 10.1016/s0022-5347(17)38337-4

[10] Aggarwal SK, Sinha SK, Kumar A, et al. Traumatic strictures of posterior urethra in boys with special reference to recurrent strictures. J Pediatric Urol. 2011;7:356–362. 10.1016/j.jpurol.2011.03.003 21527235

[11] Podestá ML. Use of the perineal and perineal-abdominal (transpubic) approach for delayed management of pelvic fracture urethral obliterative strictures in children: long-term outcome. J Urol. 1998;160:160–164. 9628640

[12] Koraitim MM. Gapometry and anterior urethrometry in the repair of posterior urethral defects. J Urol. 2008;179:1879–1881. 18353388 10.1016/j.juro.2008.01.041

[13] Pfalzgraf D, Isbarn H, Meyer-Moldenhauer WH, et al. Etiotolgy and outcome of perineal repair of posterior and bulbar urethral strictures in children: a single surgeon experience. J Pediatric Urol. 2013;9:769–774. 10.1016/j.jpurol.2012.09.007 23073040

[14] Patil UB. Long-term results of transpubic prostatomembranous urethroplasty in children. J Urol. 1986;136:286–287. 3723678 10.1016/s0022-5347(17)44843-9

[15] Das K, Charles AR, Alladi A, et al. Traumatic posterior urethral disruption in boys: experience with the perineal/transpubic approach in ten cases. Pediatr Surg Int. 2004;20:449–454. 15095103 10.1007/s00383-004-1174-y

[16] Basiri A, Shadpour P, Moradi MR, et al. Syphysiotomy: a variable approach for delayed management of posterior urethral injuries in children. J Urol. 2002;168:2166–2169. 12394751 10.1016/S0022-5347(05)64345-5

[17] Podesta M, Podesta M Jr. Delayed surgical repair of post-traumatic posterior urethral distraction defect in children & adolescents: long term results. J Pediatric Urol. 2015;11:67.e1–67.e6. 10.1016/j.jpurol.2014.09.010 25869826

[18] Badenoch AW. A pull-through operation for impassable traumatic stricture of urethra. Br J Urol. 1950;22:404–409. 14791990 10.1111/j.1464-410x.1950.tb02547.x

[19] McAninch JW. Pubectomy in repair of membranous urethral stricture. Urol Clin North Am. 1989;16:297–302. 2711548

[20] Rourke KF, McCammon KA, Sumfest JM, et al. Open reconstruction of pediatric and adolescent urethral stricture: long term followup. J Urol. 2003;169:1818–1821. 12686852 10.1097/01.ju.0000056035.37591.9f

[21] Andrich DE, Dunglison N, Greenwell TJ, et al. Long-term results of urethroplasty. J Urol. 2003;170:90–92. 12796652 10.1097/01.ju.0000069820.81726.00

[22] Vashishthha S, Sureka SK, Kumar J, et al. Predictors for recurrence after urethroplasty in pediatric and adolescent stricture urethra. J Pediatric Urol. 2014;10:268–273. 10.1016/j.jpurol.2013.08.014 24726239

[23] Singh BP, Andankar MG, Swain SK, et al. Impact of prior urethral manipulation on outcome of anastomotic urethroplasty for post-traumatic urethral stricture. Urology. 2010;75:179–182. 19854488 10.1016/j.urology.2009.06.081

[24] Helmy TE, Sarhan O, Hafiz AT, et al. anastomotic urethroplasty in a pediatric cohort with posterior urethral strictures: critical analysis of outcomes in a contemporary series. Urology. 2014;83:1145–1148. 24485997 10.1016/j.urology.2013.11.028

[25] Singla M, Jha MS, Murugagnandam K, et al. Posttraumatic posterior urethral strictures in children- management and intermediate term follow up in tertiary care center. Urology. 2008;72:540–544. 18619659 10.1016/j.urology.2008.02.078

